# Polycystic Ovary Syndrome: An Updated Overview Foregrounding Impacts of Ethnicities and Geographic Variations

**DOI:** 10.3390/life12121974

**Published:** 2022-11-25

**Authors:** Afrin Yasmin, Shubhadeep Roychoudhury, Arun Paul Choudhury, A. B. Fuzayel Ahmed, Sulagna Dutta, Filomena Mottola, Vivek Verma, Jogen C. Kalita, Dhruv Kumar, Pallav Sengupta, Adriana Kolesarova

**Affiliations:** 1 Department of Life Science and Bioinformatics, Assam University, Silchar 788011, India; 2 Department of Obstetrics and Gynecology, Silchar Medical College and Hospital, Silchar 788014, India; 3 School of Medical Sciences, Bharath Institute of Higher Education and Research (BIHER), Chennai 600126, India; 4 Department of Environmental, Biological and Pharmaceutical Sciences and Technologies, University of Campania Luigi Vanvitelli, 81100 Caserta, Italy; 5 Department of Statistics, Assam University, Silchar 788011, India; 6 Department of Zoology, Gauhati University, Guwahati 781014, India; 7 School of Health Sciences and Technology, UPES University, Dehradun 248007, India; 8 Physiology Unit, Department of Biomedical Sciences, College of Medicine, Gulf Medical University, Ajman 4184, United Arab Emirates; 9 Faculty of Biotechnology and Food Sciences, Slovak University of Agriculture in Nitra, 94901 Nitra, Slovakia

**Keywords:** polycystic ovary, PCOS, prevalence, diagnostic criteria, hyperandrogenism, hirsutism, infertility, menstrual dysfunction, ethnicity/region

## Abstract

Polycystic ovary syndrome (PCOS) is one of the most common heterogeneous conditions of the endocrine reproductive system in women of childbearing age. Hyperandrogenism and oligomenorrhea are the two core characteristics of PCOS, a complicated and multifaceted illness. The condition is also linked to several major side effects, which include type 2 diabetes, early atherosclerosis, infertility, and endometrial cancer. There are few facts and statistics available on PCOS prevalence internationally due to the significant degree of geographic and ethnic variance and inconsistency caused by different diagnosis standards. Limited (*n* = 179) explorations have been made in the context of the prevalence of this complicated illness so far, and out of these, only 55 studies have discussed its association with race and/or ethnicity. However, those studies remain restricted due to the small sample size, biased selection, and the lack of comparative studies. Variations in PCOS prevalence frequency also arise due to different diagnostic criteria, as well as racial and ethnic differences, associated lifestyle factors, and subsequent illnesses that affect the accuracy of the diagnosis. The main objective behind this systematic review is to provide comprehensive epidemiological data on PCOS that is organized geographically. This evidence-based study also provides an overview of the clinical management of PCOS to instigate further research on this complex endocrinological condition and the subsequent development of preventive treatment strategies.

## 1. Introduction

**Figure 1:**
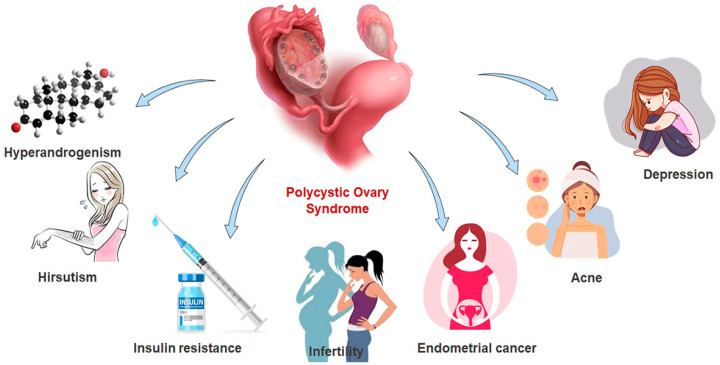
Clinical manifestations of polycystic ovary syndrome (PCOS). Complications including hyperandrogenism, infertility, hirsutism, insulin resistance, acne, and depression are markers for PCOS in women of reproductive age. Hyperandrogenism in PCOS identified by excess androgen levels in the circulation is associated with insulin-resistibility and visible features.

Polycystic ovary syndrome (PCOS) falls among the most prevalent endocrine disorders affecting about 8–13% of women of reproductive age [1,2]. PCOS is regarded as a complicated endocrinological illness associated with dysregulations of the psychological, metabolic, and reproductive systems and is a major public health concern. Due to the defining trait of PCOS, anovulation, it is the most precipitating condition affecting female fertility. Following the initial description of this condition by Stein and Levanthal in 1935 [3], numerous studies on the pathophysiology of the disease were conducted [1,2,4,5]. However, the etiology of the disorder is yet to be understood completely. Ultrasonographically, the ovaries have an increased volume with a different number of immature follicles (polycysts) [4]. The main characteristic of this syndrome is imbalanced levels of sex hormones and chronic anovulation due to increased androgen levels (hyperandrogenism), in the absence of specific adrenal and/or pituitary diseases [5]. PCOS increases the short-term and long-term risk of endometrial cancer [6], psychological problems (anxiety, depression) [7,8], pre-eclampsia [9], recurrent abortion [7], perinatal mortality [9] and possibly breast cancer and long-standing risk of obesity [10,11], type 2 diabetes, metabolic syndrome [12], hypertension [13], fetal macrosomia (baby that weighs more than 4 kg) or anomalies [14], dyslipidemia [15], cardiovascular diseases [16], thyroid [17], and hyperplasia [18]. The clinical features of this complication include infertility, hirsutism, dysfunctional uterine bleeding, pregnancy complications, irregular menstrual cycle, alopecia, and acne [7,19,20,21,22,23] as shown in Figure 1.

This syndrome is also characterized by variation in the levels of follicle-stimulating hormone (FSH), luteinizing hormone (LH), estradiol, serum androgens (testosterone and androstenedione), high anti-Mullerian hormone (AMH), prolactin, and hyperprolactinemia [5,7,23,24,25]. Moreover, insulin resistance is also associated with this disorder, where excess androgen leads to the reduced sex hormone-binding globulin (SHBG) by inhibiting its synthesis of in the liver. However, low serum SHBG levels are considered a biomarker of metabolic abnormalities and are linked with insulin resistance, hyperandrogenism, and abnormal glucose metabolism in PCOS patients, which is why the patients require long-term screening in addition to insulin-sensitizing medications such as metformin [25].

However, inconsistency has been observed in the clinical symptoms of PCOS based on age (adolescents to post-menopausal phase), accompanied endocrine or metabolic disorders, lifestyle factors, environmental factors, genetics and, also on ethnicity, race, and geographic location. The prevalence of PCOS in different ethnic backgrounds varies greatly, particularly due to the high degree of inconsistency in the sample size and the diagnostic criteria used in previous studies [7,26]. Indigenous women of different regions appear to be among the high-risk populations in terms of PCOS prevalence and complications, as compared to other communities [26,27,28]. The 2018 PCOS guidelines from the National Health and Medical Research Council (NHMRC), Australia, recommended the inclusion of ethnic variation for better management of the disease. According to the guideline, health professionals and scientists should consider ethnic variation in the manifestations of PCOS, including differences in hirsutism patterns [19,29], acanthosis nigricans, and in metabolic sequelae, including obesity and insulin resistance, while consideration should be predominantly given to South Asian and high-risk ethnicity and for adolescent-specific ranges for determining the frequency of risk assessment [28,30]. The objective of this systematic review is to provide comprehensive epidemiological data on PCOS that is organized geographically. The rationale behind the present study was to highlight the available facts and statistics on PCOS prevalence internationally and the significant degree of geographic and ethnic variance and inconsistency caused by different diagnosis standards.

## 2. Materials and Methods

### 2.1. Eligibility Criteria

A complete literature survey was conducted to acknowledge the very recent and informative data on PCOS with respect to the ethnicity and geographical location prevailing. All the women of the child-bearing age group presenting symptoms of PCOS-based case–control, cross-sectional, cohort, or observational-type published studies were selected to ascertain the research gap and write this piece of systematic review. Furthermore, the published articles where the prevalence of PCOS accounting for different ethnic backgrounds and regions was mentioned or calculated were included in the study. However, the articles following different diagnostic criteria other than Rotterdam, 2003; National Institute of Health (NIH), 1990 and ESHRE/ASRM, 2006 were excluded from the study.

### 2.2. Search Strategy and Data Extraction

The search approach followed in this systematic review was performed according to the guideline of the Preferred Reporting Items for Systematic Reviews and Meta-Analyses (PRISMA) [31]. Through this systematic review, we have tried to ascertain the most recent and relevant published studies on the prevalence of PCOS. Hence, we limited the time threshold from January 2000 to September 2022 inclusion in the PubMed database. Further, filters were also applied for more relevant results, such as the language selected being English and those that were meta-analysis, clinical studies, controlled and randomized clinical trials, observational studies, case reports, reviews, and systematic reviews excluding books, documents, and editorials. Moreover, the data extraction was performed following sample size, prevalence, criteria, age, hirsutism, serum androgen level, type of menstrual imbalances, fertility status, ovarian morphology, geographic region, ethnicity of the participants, etc. Further, the search string was designed with the help of appropriate Boolean operators to retrieve the relevant published data. Bibliographies of relevant studies were also searched to identify additional sources. Keyword string that was prepared for relevant data search was: (Polycystic ovary syndrome) OR (PCOS) OR (Stein-Leventhal syndrome) OR (Hyperandrogenism) OR (Androgen Excess) AND (Prevalence) AND (Ethnic*) AND (Geographic*) AND (PCOS Diagnostic Criteria) OR (Polycystic ovary syndrome Diagnostic Criteria).

### 2.3. Clinical Diagnosis and Criteria

PCOS mainly affects women of reproductive age based on specific clinical signs. In addition to hereditary variables, the incidence ratio of PCOS greatly varies by race, ethnicity, and locality. PCOS is diagnosed in 80% of patients with oligomenorrhea and up to 30% to 40% of patients presenting with primary or secondary amenorrhea, while up to 70% of women remain undiagnosed [32]. In fact, PCOS is a broad concept, and there are three principal diagnostic characteristics—menstrual dysfunction, clinical hyperandrogenism, and chronic anovulation. The clinical symptoms of hyperandrogenism include androgenic alopecia, acne, hirsutism, and male-like features that occur in more than 80% of women with PCOS [33]. Additionally, as the symptoms are associated with hormonal imbalances, variations in LH, FSH, prolactin, estrogen, and serum androgen levels, such as testosterone or androstenedione, are perfectly typical. Increased levels of the LH/FSH ratio are indicated by biochemical and hormonal evaluations [34].

Previously, there were three sets of consensuses for the diagnosis of PCOS. Based on clinical, biochemical, and ultrasound image results, each group of consensuses has slightly different criteria from the others [35] (Table 1). The first consensus proposed by the NIH in 1990 involves the symptoms of -(a) hyperandrogenemia/ hyperandrogenism, and (b) ovulation disorder (amenorrhea/oligomenorrhea) in the absence of non-classical adrenal hyperplasia [36]. The second one was introduced by the Fertility and Embryology Association of Europe and the American Fertility Society at Rotterdam Conference (Rotterdam criteria, ESHRE/ASRM-Sponsored PCOS Consensus Workshop Group Fertility and Sterility, 2003) [37]. They considered any of the following two characteristics for diagnosis of PCOS—(a) oligo-ovulation or anovulation below 9 cycles per year, (b) clinical or biochemical signs of hyperandrogenism or elevated serum androgen levels, and (c) the occurrence of polycystic ovaries with pelvic ultrasound (considered if more than 12 follicles measure 2 to 9 mm and ovarian volume is greater than 10 cm^3^). Furthermore, the exclusion characteristics that can cause and interfere with chronic anovulation and androgen excess include congenital adrenal hyperplasia (classical and non-classical form), hyperprolactinemia/hyperthyroidism and Cushing’s syndrome (secretory ovarian tumor of adrenal androgens) [37].

Thirdly, the Androgen Excess and PCOS Society (AE-PCOS—The Thessaloniki ESHRE/ASRM- Sponsored PCOS) introduced criteria in 2006 [6,21] according to which the presence of hyperandrogenism/androgen excess and oligo- and/or anovulation or polycystic ovaries for PCOS diagnosis was considered [20,23,35,38].

This criterion primarily assessed the disease as an androgen excess problem since this is one of the main features of PCOS. Hyperandrogenemia, clinical hyperandrogenism, menstrual or ovulatory dysfunction, and polycystic ovaries are further symptoms of the androgen excess condition. Later, this criterion reported that the resulting phenotypes with such combined traits may contain insulin resistance and other metabolic problems and did not necessarily constitute PCOS individually [20,23,35,36,37,38].

The most recent inclusion of diagnostic criteria to maintain the compatibility for PCOS research was globally introduced by NHMRC, Evidence-based Methodology Workshop Panel on Polycystic Ovary Syndrome in 2012 [19,39] addressed the benefits and drawbacks of the existing diagnostic criteria and further recommended the importance of ESHRE/ASRM 2003 criteria in broader terms including the identification of phenotypes and sub-phenotypes, as mentioned in Table 2. In addition, the NHMRC guidelines on PCOS-2018 endorsed the widely accepted Rotterdam criteria with minimal changes; for example, ultrasound imaging is not required if the patient has menstrual dysfunction in addition to hyperandrogenism, but it may be essential for phenotypic patterns [19,39]. It is apparent that the diagnostic criteria for PCOS are being revised periodically, and the amendments are made according to the understanding of its etiology [40].

## 3. Results and Discussion

### 3.1. Search Results

**Figure 2:**
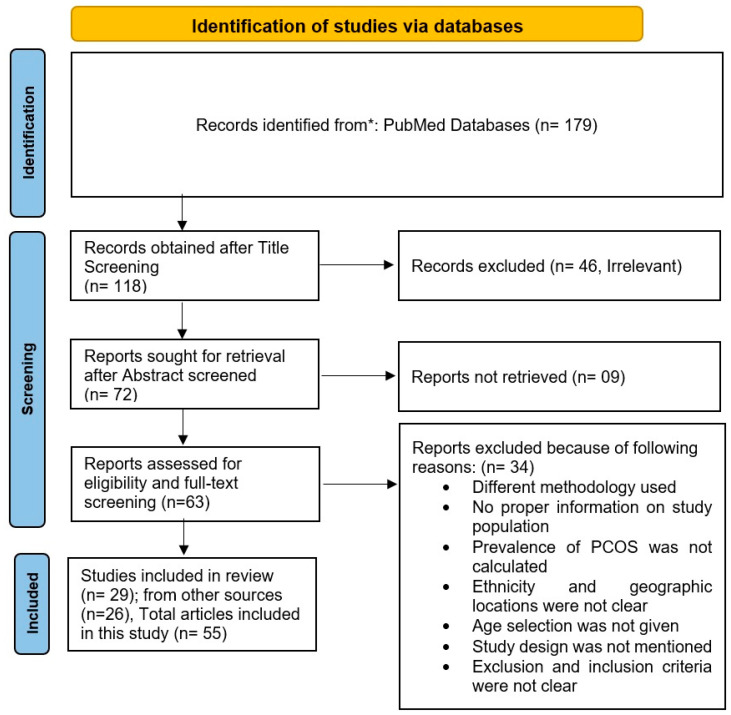
Flow chart depicting the literature-survey process based on the PRISMA guideline.

After an in-depth search in the databases with mentioned strings and Boolean operators, a total of 179 resultant articles were identified. Of these, 61 articles were removed because of irrelevancy, following which 118 published articles were recognized for further screening, whose titles and abstracts were assessed in a fitly way, followed by the removal of irrelevant content. Furthermore, only 63 articles found to be appropriate for this article were retrieved, reviewed, and screened for full text. After proper refinement and understanding, 29 articles were taken on execution and selected for inclusion in this systematic review article. A PRISMA 2020 flow diagram is provided in Figure 2, depicting the articles included and excluded in the study. For ease of understanding, the PRISMA 2020 checklist and abstract checklist are given as non-published materials. Furthermore, for more relevant data on ethnicity, geographical region-based studies, and prevalence of PCOS estimation, additional bibliographies were also searched with all possible combined keywords. After the title, abstract, and full-text screening of the most suitable articles, a total of the remaining 26 articles were retrieved and included in this study for providing the most notable and facts-enhancing information through this systematic review.

### 3.2. Epidemiology in Different Ethnicities

There are few studies that reported the prevalence of PCOS, and they vary greatly in terms of sample size, ethnicity, race, and geographical location [19,29,41]. It is approximated that as high as 105 million women of childbearing age are affected by PCOS worldwide, while in 72–82% of cases, hyperandrogenism is mainly responsible for PCOS development [35].

Diagnostic criteria are also believed to impact the prevalence of PCOS [29]. Studies showed a significant difference in the clinical symptoms observed across geographical locations among different ethnic groups [29,40,41,42]. One of the largest studies on the prevalence of PCOS among the geographically diverse American population showed a higher prevalence of the disease in the southern part of the USA by 47.5% in comparison to the rest of the country [41]. Studies on the prevalence of PCOS among different ethnicities have been conducted among the Caucasian population, mainly in Australia, USA, and Spain [27,29,41,42,43].

### 3.3. Prevalence in Europe, America, and Australia

In Spain, Asuncion et al. assessed the prevalence in an unbiased manner with a study sample of 154 women in Madrid by means of NIH diagnostic consensus. The study was not completely randomized and reported an incidence rate of 6.5% [43].

Lauritsen et al. (2014) performed a prospective, cross-sectional study in Denmark and found a prevalence of PCOS of 16.6% according to the Rotterdam criteria [24]. The study included 863 women aged 20–40 years. However, the main result indicated a significant decrease in the prevalence of PCOS by 33.3% in women <30 years to 14.7% in women aged 30–34 years, while 10.2% in women ≥35 years (*p* < 0.001). A total of 53.5% fulfilled the criterion for polycystic ovaries. In addition, AMH or age-adjusted AMH Z-score was found to be a reliable marker of polycystic ovaries in women with PCOS [24].

In addition, a cross-sectional study of 192 women of reproductive age (17–45 years) living on the Greek island of Lesbos was conducted by Diamanti-Kandarakis et al. (1999) and found an estimated PCOS prevalence of 6.77%. They also suggested that the disorder is associated with certain metabolic abnormalities such as obesity and hyperinsulinemia [44].

Sanchon et al. (2012) conducted a multicenter survey that included 592 consecutive premenopausal women (393 from Madrid, Spain, and 199 from Bologna, Italy) reporting spontaneously for blood donation. Before donation, the team conducted clinical and biochemical phenotyping for androgen excess disorders and reported that hyperandrogenism, hirsutism, and acne were equally frequent [12.2% prevalence; 95% confidence interval (CI): 9.5–14.8%], whereas alopecia was uncommon (1.7% prevalence, 95% CI: 0.7–2.7%). However, functional disorders of androgen excess, PCOS, and idiopathic hirsutism were equally frequent (5.4% prevalence, 95% CI: 3.6–7.2), followed by idiopathic hyperandrogenism (3.9% prevalence, 95% CI: 2.3–5.4) [45].

Similarly, in 2010, March et al. conducted a large cohort study in Adelaide, Australia, with 728 Caucasian subjects using all three sets of criteria. The study assessed a prevalence of 11.9 *±* 2.4%, 10.2 *±* 2.2%, and 8.7 *±* 2.0% PCOS according to the Rotterdam, the AES criteria, and the NIH, respectively. Despite inconsistencies, it remains the only large community-based retrospective study to determine the PCOS prevalence rate in the homogeneous Caucasian population in that particular geographical region [42].

In Brazil, a study was conducted using the Rotterdam consensus and reported a prevalence of 8.5% (95% CI: 6.8 to 10.6) in the first such initiative of epidemiological investigation on PCOS among Latin American women [46].

The rate of PCOS in the Hispanic community was also studied by Moran et al. in 2010 according to the NIH criteria and reported a prevalence of 6.0% (95% CI: 1.9–10.1%), while by the Rotterdam criteria, it was 6.6% (95% CI: 2.3–10.9%) among the Mexican women residing in Mexico City [47]. Previously, Goodarzi et al. found a prevalence of 13% in Mexican-American women residing in Los Angeles [48]. Furthermore, it has also been reported that Hispanic women have a significantly higher rate of androgen index, hirsutism, and hyperglycemia. Such studies possibly indicate a higher prevalence of PCOS among Mexican-American women, which may be associated with their lifestyle [48]. However, indigenous Australian women showed a significantly higher prevalence of 26%, along with higher rates of obesity, hirsutism, and type 2 diabetes compared to Caucasians. This may be attributed to the gradual change of the indigenous women from a hunter-gatherer way of living to a more sedentary lifestyle with a nutritionally poorer diet [27].

### 3.4. Prevalence in Iran

Jalilian et al. reviewed the prevalence of PCOS and its associated complications among Iranian women using all three criteria and reported that the prevalence of PCOS at 19.5%, 6.8%, and 4.41% according to the Rotterdam criteria, NIH criteria, and ultrasound investigation, respectively. In addition, the prevalence of hirsutism was assessed as 13%, androgenic alopecia at 9%, acne at 26%, menstrual abnormalities at 28%, infertility at 8%, and obesity at 19%. The study concluded that the prevalence of PCOS in Iranian women is not high as compared to other communities residing there. However, associated health complications, such as infertility and cardiovascular diseases (CVD), were indeed high and needed attention [49]. The prevalence of PCOS among Qatari women was studied by Dargham and his co-workers in 2017 among 3017 subjects aged 18 to 40 years. The NIH guidelines have identified PCOS in 720 of the patients who underwent sex-hormone-binding globulin (SHBG) and testosterone examinations. The prevalence of PCOS was reported to be 12.1% in their study [50].

### 3.5. Prevalence in Asia

Recently, Kim and Choi addressed the phenotypic and genetic patterns of PCOS in Asian women. They reported that East Asian patients have a hirsutism score cutoff [according to the Ferriman–Gallwey (F–G) score] lower than that of the Caucasian population, a higher degree of insulin resistance, a lower index of metabolic syndrome, and a lower body mass index (BMI) [51]. Moreover, when Caucasian women were compared to their East Asian counterparts in terms of irregular menses (IM/PCO sub-group), they did not share a common phenotype. Whereas South Asian patients showed high symptoms of hirsutism, early onset of symptoms, increased insulin resistance, and other metabolic risk factors when measured to Caucasians. The study also suggested that genetic factors play a crucial role in the pathogenesis of the symptom. Genome-wide association studies on different ethnicities revealed that both Asian and Caucasian women share similar genetic risk factors [51]. The dysregulation of androgen synthesis, LH and androgen-receptor gene, and genes involved in insulin action and secretion that may be triggered by genomic variants have been linked to hyperandrogenism and environmental risk factors, such as sedentary lifestyle and dietary habitats [7]. Ding et al. used the random effect model to compare the four major contrasting ethnic groups, such as Middle Eastern, Caucasian, Chinese, and African-American females. The study reported the lowest prevalence among Chinese women (5.6%), followed by Caucasians (5.5%), Middle Eastern (6.1%), and Black women (7.4%) by NIH criteria [52]. Furthermore, in Asia, there is a large number of ethnic groups, which may account for the possible variation in the prevalence and symptoms of PCOS. Kumarapeli et al. conducted a large community-based study including over 3000 women between the age of 15 to 39 years in Sri Lanka, and reported a prevalence of 6.3% based on the Rotterdam criteria. However, Asian women were found to have a lower hirsutism index as compared to the Caucasian population [53]. Notwithstanding, Sri Lankan women have been suspected of having a higher rate of prevalence of PCOS as compared to the Caucasian population, possibly due to a higher association with type 2 diabetes [53,54]. Moreover, Japanese women showed lower levels of hirsutism, obesity, and insulin resistance apart from the prevalence of PCOS when compared to Caucasians and other major groups [28].

### 3.6. Prevalence in India

A few studies have been conducted in India to determine the incidence of PCOS and associated symptoms. After ruling out thyroid dysfunction, adrenal hyperplasia, and hyperprolactinemia, Deswal and his colleagues performed large-scale research in Haryana among women of reproductive age from both urban and rural backgrounds who reported having irregular menstruation, hyperandrogenism, and PCOS. The research, which used the Rotterdam criteria, found a prevalence of PCOS of 4.21%. The results also revealed that women living in urban regions had a greater risk of PCOS than women living in rural areas. Urban residents may have a higher prevalence of PCOS because of their sedentary lifestyle and eating habits, which may ultimately exacerbate the risk factors for this chronic condition [55]. Similar to this, Bharati & coworkers earlier carried out cross-sectional research on a randomly selected population to ascertain and contrast the prevalence of PCOS in both rural and urban women populations in Chennai, Tamil Nadu, India. According to the Rotterdam criteria, a prevalence rate of 6% was determined. The study also found that urban women are 0.1 times more likely than rural women to be at risk for PCOS. Additionally, it was shown that stress plays a significant part in triggering the symptoms and contributing to the development of PCOS [56]. As shown in Table 3, Indian women evidently had a higher prevalence of PCOS when compared to their US counterparts [56].

## 4. Management

**Figure 3:**
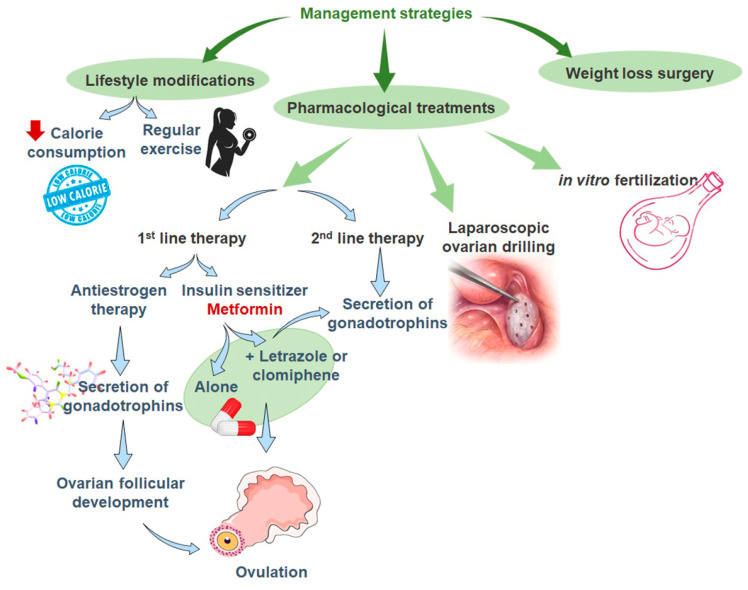
Clinical approaches for polycystic ovary syndrome (PCOS) management and therapy.

PCOS is generally associated with hormonal imbalance that leads to multiple metabolic complications through variations in the concentration levels of serum androgen and estrogen [1,19,20,57]. The main goal of PCOS management should be to identify hormonal dysregulations [19,58]. Additionally, it has been noted that PCOS therapy may differ for patients from various ethnic and regional origins, pointing to the necessity for individualized treatment plans for improved PCOS control [29,42,59]. The basic management strategy of PCOS comprises lifestyle modification as the first-line of therapy that includes decreased daily calorie consumption, regular physical exercise followed by pharmacological treatment, and weight loss surgery [19,58,60] or sometimes bariatric surgery, as recommended for morbid obesity [61,62], as shown in Figure 3. Pharmacological treatment includes ovulation induction involving anti-estrogen therapy with clomiphene citrate, which is also considered the standard first-line therapy for anovulatory PCOS that enhances the secretion of gonadotropins and promotes ovarian follicular development [58,60,61]. On the other hand, insulin sensitizer metformin can also be used for ovulation induction, either alone or in combination with letrozole or clomiphene citrate, for enhanced effects [63]. Letrozole has recently been recommended as a first-line pharmacological treatment for ovulation induction, and it may soon replace the previous theory of clomiphene citrate use, however use of combination of both letrozole and clomiphene citrate could provide better result compared to letrozole alone [64]. In addition, gonadotropins form the second-line therapy for ovulation induction in patients with failed ovulation and are resistant to oral agents [64,65]. Another management strategy known as laparoscopic ovarian drilling has similar effectiveness as gonadotropins in the treatment of clomiphene-citrate-resistant PCOS. It consists of the induction of ovulation by the laparoscopic perforation of membranes surrounding the ovary, and it is more effective in patients with high LH index, women who need laparoscopic assessment of their pelvis, or patients who are unable to monitor gonadotropin therapy at regular intervals. However, patients may also be referred to assisted reproductive technology (ART) when they fail to respond to the first-line or second-line treatments for ovulation induction [66]. However, several candidate genes have already been identified via genome-based studies to improve screening and treatment facilities for PCOS that show more potential and efficiency [7,42,63]. However, much more is needed before it can be applied in the field of clinical practice. Moreover, ethnicity-specific strategies for PCOS diagnosis are highly required and preferred to recognize the anthropometric threshold variations and phenotypic expressions for better treatment and diagnosis in high-risk ethnic communities [29,66]. Although, the major alarming issue for women with symptoms of PCOS is the long-term metabolic risk factors [67]. Hence, the clinical management of the disorder is suggested to be immediately performed after a diagnosis confirms in order to avoid the severity and further complications [68].

## 5. Conclusions and Future Perspectives

PCOS is a common endocrine disorder among women of reproductive age, and its prevalence may vary depending on the different associated factors, which include ethnicity, race, community, and geographical region. This study aimed to review the pattern and the prevalence of PCOS based on geographical regions and variations. In addition, it also provides the researchers and clinicians with a concise and updated overview of the pathophysiology, diagnostic criteria, and management of PCOS, as well as the knowledge gap, which may encourage further interventions in this direction. Based on this study, we may conclude that the prevalence of PCOS across the USA, Spain, Brazil, Mexico, Iran, Africa, and Asia lies between 6% and 9%. Qatari women showed a higher prevalence of PCOS than the Caucasian population, while Chinese women were suggested to have the lowest prevalence when compared using the NIH criteria. This study reveals that there are moderate to high racial or ethnic influences observed on the prevalence of PCOS. The variation is quite negligible, which could be due to the low accuracy and comparability amongst the study design, biased selection of the sample, size of the sample, different parameters in the criteria, limited research, and the literature. Depending on the diagnostic consensuses, PCOS prevalence may also vary. Recent evidence significantly showed a difference in ethnicity, phenotype, and morbidity among PCOS patients. Due to numerous postulated causes of PCOS, there is a considerable dearth of knowledge regarding the risk factors and diagnosis. Low to moderate research on PCOS, as revealed by the available literature, indicates the need for expanding the research base based on the prevalence and impact on a substantive proportion of the population, as the existing data do not specifically portray the prevalence and etiology of the disorder, including the hormonal disturbances among such women of childbearing age. Moreover, although the correlation between PCOS and female infertility is known, at present, the mechanisms underlying PCOS onset in women are not yet fully understood given the variable symptoms, so further investigation may be helpful in exactly revealing the extent to which PCOS contributes to female infertility. Further research and epidemiological information are needed for a better understanding of the diagnostic criteria and the natural course of the disorder, as well as to validate any strength of true linkage with comorbid syndrome or disorder. The data currently available are insufficient to draw conclusions or calculate the precise prevalence. The known facts on PCOS and its incidence rate across different geographical regions are not conclusive enough to verify the significant differences in the rate of prevalence of PCOS across ethnic groups and geographical regions varying with incidence rates. Moreover, there is not enough information available regarding the diagnostic criteria of PCOS and its association with infertility. This detailed review provides an evidence-based update on the understanding of the prevalence of PCOS based on ethnicities and geographical locations.

## Figures and Tables

**Table 1:** Summary of all the diagnostic consensus for polycystic ovarian syndrome (PCOS).

**Diagnostic Consensus**	**National Institutes of Health (NIH), 1990** [36]	**Rotterdam, 2003** [37]	**AE-PCOS Society, 2006** [20,23]	NHMRC-Evidence-Based Methodology Workshop Panel on PCOS, 2018 [19]
Symptomatic characteristics	i. Clinical or Biochemical hyperandrogenism, ii. Ovulation disorder (amenorrhea/oligomenorrhea) in the absence of non-classical adrenal hyperplasia	i. Androgen excess/hyperandrogenism ii. Oligo-ovulation or anovulation, Polycystic appearance of the ovaries on ultrasonography	i. Androgen excess ii. Ovarian dysfunction	i. Hyperandrogenism ii. Oligo-and/or anovulation iii. Polycystic appearance of the ovaries on ultrasonography
Criteria considered	Both criteria are considered	Considers any two of three criteria	Both criteria are required	i. Any two of three criteria are considered to identify sub-phenotypes ii. Polycystic ovaries on USG are required.

AE-PCOS Society—The Androgen Excess and Polycystic Ovary Syndrome Society; NHMRC—National Health and Medical Research Council; USG—Ultrasonography.

**Table 2:** Classification of polycystic ovarian syndrome (PCOS) phenotypes.

Parameters	Phenotype A	Phenotype B	Phenotype C	Phenotype D
HA	+	+	+	_
OD	+	+	_	+
PCOM	+	_	+	+

HA—androgen excess/hyperandrogenism, OD—ovulatory dysfunction, PCOM—polycystic ovary morphology.

**Table 3:** Important epidemiologic studies on polycystic ovary syndrome (PCOS) summarized based on type of study and different ethnicities and regions.

Geographic Region	Ethnicity	Sample Size	Objectives	Findings	Consensus	Reference
Cross-Sectional Study						
Spain	Caucasian	154 women	Assessment of PCOS prevalence in an unbiased manner	PCOS prevalence rate of 6.5%	NIH Criteria	[43]
Mexico	Hispanic	150 women	Assessment of PCOS prevalence	PCOS prevalence rate in Mexican women is similar to other populations but lower than reported in Mexican American women	NIH and Rotterdam criteria	[47]
Sri Lanka	South Asian	3030 women	Assessment of PCOS prevalence and symptoms, large community-based	Oligo/amenorrhea and polycystic ovaries (91.4%) oligo/amenorrhea and hirsutism (48.3%)	Rotterdam criteria	[53]
Retrospective Study						
Australia	Caucasian	728 women	Assessment of PCOS prevalence with large cohort study	The Rotterdam and AES prevalence estimates were up to twice that obtained with the NIH criteria	NIH, Rotterdam, and the AES criteria	[42]
Meta-analysis						
Iran	Iranian	30 studies	Reviewed the prevalence of PCOS and its associated complications	PCOS prevalence rate in Iran is not high	NIH criteria, Rotterdam criteria & Ultrasound investigation	[49]
Different sources and regions	Caucasian, Middle Eastern, Chinese, and African American	42 studies	Investigation four major contrasting ethnic groups	PCOS prevalence rate in Chinese women was lowest than in black woman	NIH, Rotterdam, and the AES criteria	[29]

NIH—National Institute of Health; AES—The Androgen Excess Society.

## Data Availability

Not applicable.
